# Modeling neurovascular dysfunction in Alzheimer’s disease using an isogenic brain-chip model

**DOI:** 10.1186/s12987-025-00708-y

**Published:** 2026-01-06

**Authors:** Andrew N. Shen, Katelin S. Matazel, W. Drew Gill, Lorna Ewart, Randy S. Daughters, Hector Rosas-Hernandez

**Affiliations:** 1 Division of Neurotoxicology, National Center for Toxicological Research/Food and Drug Administration, Jefferson, AR 72079 USA; 2 Emulate Inc., 27 Drydock Avenue, Boston, MA 02210 USA

**Keywords:** Neurovascular unit, Alzheimer’s disease, Organ-chips, Human induced pluripotent stem cells, Blood-brain barrier, Disease modeling

## Abstract

**Background:**

The pathology of Alzheimer’s Disease (AD) is characterized by aggregates of amyloid beta (Aβ) peptides and neurofibrillary tau tangles. Increased blood-brain barrier (BBB) permeability and reduced Aβ clearance, which signal neurovascular dysfunction, have also been proposed as early markers of AD. Despite intense scrutiny, the mechanisms of AD remain elusive and novel treatments that address core symptoms of dementia are limited. New alternative methods (NAMs) aim to develop in-vitro translational models that recapitulate human pathology more accurately than previous models and could contribute to the development of new therapies.

**Methods:**

Here, we developed a NAM model of the cortical neurovascular unit (NVU) using brain cells derived from human induced pluripotent stem cells (hiPSCs) from a patient with AD and a healthy individual. Differentiated neurons, astrocytes, pericytes, microglia, and brain-like microvascular endothelial cells were cultured in a microphysiological system to create a brain-chip model to evaluate NVU-related endpoints.

**Results:**

Compared to control, AD brain-chips had reduced claudin-5 and ZO-1 expression and increased paracellular permeability. AD brain-chips also had decreased activity of the efflux transporter P-glycoprotein (P-gp), but its expression was unchanged. In AD brain-chips, levels of Aβ42, total tau, and p-tau 181 were decreased in protein lysates from the brain channel, while levels of total tau and p-tau 181 were increased in protein lysates from the vascular channel. Finally, AD brain-chips had increased levels of the proinflammatory markers IL-6 and MCP-1 in effluent from both brain and vascular channels.

**Conclusion:**

In this brain-chip model, we showed Aβ-independent NVU dysfunction that was related to neuroinflammation and vascular tau accumulation. This study demonstrates the utility of the brain-chip model to evaluate changes in NVU functions induced by AD-like pathology and highlights donor-specific responses associated with the use of hiPSC-derived models.

**Supplementary Information:**

The online version contains supplementary material available at 10.1186/s12987-025-00708-y.

## Introduction

Alzheimer’s disease (AD) pathology is characterized by the presence of excess free and aggregated amyloid beta (Aβ) peptides and by intracellular neurofibrillary tangles that contain hyperphosphorylated and misfolded tau protein [1]. The brain vasculature is also impaired in AD, which may contribute to or exacerbate the underlying pathophysiology of the disease [2, 3]. This impairment can manifest as increased blood-brain barrier (BBB) permeability to neurotoxic plasma-bound molecules [2, 4–6] and impaired brain-to-blood clearance of Aβ peptides [7].

To better understand the pathology of AD, several preclinical models have been developed [8]. While familial cases of AD account for a small fraction of total cases [9], preclinical rodent models overwhelmingly rely on overexpression of human mutations associated with familial AD, including amyloid precursor protein (APP), presenilin-1 (PSEN1) and presenilin-2 (PSEN2) [10, 11]. Recent attempts to model the pathophysiology of sporadic AD have used APOE4 allele expression, which is the strongest genetic risk factor to develop AD [12, 13]. Although rodent models have been invaluable in gaining insight into mechanisms of AD pathology, few capture the complete pathological features observed in human patients with AD [14]. Innovative approaches to produce better models of AD pathology and increase translational value includes the use of human induced pluripotent stem cells (hiPSCs) derived from patients with AD [15–17].

Patient-derived hiPSCs can be differentiated into multiple brain cell types affected in AD, including neurons [18], astrocytes [19–22], microglia [23], pericytes [24] and brain microvascular endothelial-like cells (iBMVECs) [25]. These differentiated cells express pathological characteristics of the individuals from which they were obtained, and can be an extremely valuable tool in the understanding of AD. Neurons differentiated from those hiPSCs produce, accumulate, and secrete Aβ and tau in larger amounts than neurons differentiated from hiPSCs from healthy individuals, replicating key characteristics of human AD pathology [20, 26–31]. Additionally, astrocytes [19–22], microglia [32, 33], pericytes [24, 34] and iBMVECs [35, 36] derived from hiPSCs from patients with AD present with hallmarks of AD pathophysiology respective to each cell type. These studies highlight the utility of using patient-derived hiPSCs to model different characteristics of AD pathology, including neurovascular dysfunction.

To help account for the complexity of in vivo systems, novel in vitro systems, like microphysiological systems (MPS) or organ-chips, have recreated key characteristics of human brain physiology, like blood flow and mechanical shear forces [37–44], which enable cells to acquire physiological or pathological functions that cannot be achieved in static 2D cultures [45, 46]. Coupling the use of organ-chips to model human physiology, with brain cells derived from hiPSCs that present pathological hallmarks of AD, can lead to the development of better models that closely recapitulate AD pathology.

Here, we differentiated cells of the NVU from hiPSCs obtained from a patient with AD and from a healthy individual and cultured them on a brain-chip under flow conditions to develop a model of the NVU. In these patient-specific AD brain-chips, we observed NVU dysfunction that was related to vascular tau accumulation but seemingly independent of Aβ production. This study demonstrates the utility of a hiPSC-derived isogenic brain-chip in modeling patient-specific characteristics of AD pathology.

## Methods

### Cell lines

The hiPSC line MC0117 (APOE ε3/ε3, apparently healthy individual) was obtained from the Mayo Clinic’s Neuroregeneration Lab within the Center for Regenerative Medicine. The hiPSC line AG27609 (APOE ε4/ε4, late-onset AD patient) was obtained from the Coriell Institute for Medical Research/National Institutes of Aging’s Aging Cell Repository. Colonies of hiPSCs were maintained in 6-well plates coated with Matrigel (Corning) in complete mTeSR Plus media (StemCell Technologies), with media changes performed every other day. Cells were passaged at a 1:6 ratio, when 80% confluency was reached, using ReLeSR passing reagent (StemCell Technologies).

### Differentiation of iBMVECs

Differentiation of hiPSCs into iBMVECs was performed following a previously published protocol [25]. Briefly, hiPSCs colonies were singularized with Accutase (Thermo Scientific) and then plated into Matrigel-coated T75 flasks at a density of 25,000 cells/cm^2^ in complete mTeSR Plus media, in presence of 10 µM of the ROCK inhibitor Y-27632 dihydrochloride (StemCell Technologies) for 24 h. Then, media was changed to DeSR1 media that consisted of DMEM/F12 media (Gibco), 1% non-essential amino acids (Corning), 1% glutamine (Corning), 0.1 mM β-mercaptoethanol (Gibco), and 6 µM of the GSK3β inhibitor CHIR99021 (StemCell Technologies). Cells were cultured on DeSR1 media for 24 h and then changed to DeSR2 media (DeSR1 media supplemented with 1X B27 (Gibco) and without CHIR99021). Cells were cultured in DeSR2 media for 5 days with daily media changes. Then, cells were cultured for 3 days in hECSR1 media that consisted of human endothelial cell serum free media (hECSFM) (Gibco), 1% B27, 20 ng/mL basic fibroblast growth factor (bFGF) (Millipore), 10 µM retinoic acid (Sigma), with one media change performed after 48 h. Media was then changed to hECSR2 media that consisted of hECSR1 media without bFGF and retinoic acid and supplemented with 1% penicillin/streptomycin (Gibco) for 24 h. Then, differentiated iBMVECs were singularized and plated into T75 flasks coated with 400 µg/mL human collagen IV (Sigma Aldrich) and 100 µg/mL fibronectin (Sigma Aldrich) in hECSR2 media supplemented with Y-27632 dihydrochloride for 2 hours, and then media was replaced with hECSR2 and maintained in culture for 24 to 48 h.

### Differentiation of pericytes

Pericytes were differentiated following a previously described protocol [36]. Briefly, hiPSCs were singularized with Accutase, plated into Matrigel-coated 100 mm dishes at a density of 25,000 cells/cm^2^ in complete mTeSR Plus media, supplemented with 10 µM Y-27632 and maintained in culture for 24 h. Cells were then cultured for 3 days in N2B27 media, consisting of 1:1 Neurobasal (Gibco) and DMEM/F12 media, 1% glutamine, 1% penicillin/streptomycin, 1X B27 and 1X N2 (Gibco), and supplemented with 25 ng/mL bone morphogenic protein 4 (BMP4) and 8 µM CHIR99021. After that, cells were cultured for 48 h in N2/B27 media supplemented with 10 ng/mL platelet-derived growth factor BB (PDGF-BB) (Peprotech), and 2 ng/mL Activin A with daily media change. Differentiated pericytes were then maintained in N2/B27 media for 24 to 48 h.

### Differentiation of microglia

Microglia were differentiated from hiPSC adapting previously described methods [47, 48]. In short, hiPSCs were singularized with Accutase and plated at a density of 3 × 10^6^ cells per well on AggreWell 800 6-well plates (StemCell Technologies) in embryoid body media that consisted of mTeSR plus media supplemented with 10 µM Y-27632, 50 ng/mL BMP4, 20 ng/mL stem cell factor (Peprotech), and 50 ng/mL vascular endothelial growth factor 121 (Peprotech). Plates were spun down at 300 g for 3 min and maintained in culture for 2 days, before performing half media change. On day 4, embryoid bodies were collected and transferred to Matrigel-coated 6-well plates in 3 mL of hematopoietic media, consisting of X-VIVO 15 media (Lonza), supplemented with 1% glutamine, 1% penicillin/streptomycin, 55 µM β-mercaptoethanol, 100 ng/mL Macrophage Colony Stimulating Factor (Peprotech), and 25 ng/mL IL-3 (Peprotech). Cells were maintained in culture for 15 days, with half media changes performed every 5 days. After 15 days in culture, primitive macrophage precursor cells (PMPs) are produced and released to the media. On day 15, PMPs were harvested and plated in 6-well plates in RMPI 1640 media at a density of 180,000 cells/cm^2^. Cells were allowed to attach for 1 h and then media was replaced with complete microglia media that consisted of RMPI 1640 media supplemented with 1% glutamine, 1% penicillin/streptomycin, 100 ng/mL IL-34 (Peprotech), and 10 ng/mL Granulocyte-Macrophage Colony-Stimulating Factor (Peprotech). PMPs were cultured for 10 days with media change performed every other day. Differentiated microglia were maintained in microglia media for up to 10 days.

### Neural progenitor cells differentiation

Neural progenitor cells (NPCs) were differentiated from hiPSCs using the STEMdiff SMADi Neural Induction Kit (StemCell Technologies). Singularized hiPSCs were plated in Matrigel-coated 6-well plates at a density of 2.5 × 10^5^ cells/cm^2^ in STEMdiff neural induction media (NIM) + SMADi supplement and 10 µM Y-27632. Daily media changes were performed for 6 days using STEMdiff NEM + SMADi. On day 6, cells were passaged to Matrigel-coated 6-well plates at a density of 2.0 × 10^5^ cells/cm^2^ in STEMdiff NIM + SMADi supplement and 10 µM Y-27632. Daily media changes were performed for another 6 days using STEMdiff NEM + SMADi. Cells were passaged again on day 12 following the same procedure and cultured for an additional 6 days. On day 18, NPCs were passaged into Matrigel-coated 6-well plates at a density of 1.25 × 10^5^ cells/cm^2^ in neural progenitor media (StemCell Technologies). NPCs were maintained in culture for 7 days with daily media change.

### Differentiation of neurons

Neurons were differentiated from NPC using the STEMdiff forebrain neuron differentiation kit (StemCell Technologies). Briefly, NPCs were cultured in 6-well plates coated with 15 µg/mL poly-L-ornithine (PLO) (Sigma) and 5 µg/mL laminin (Sigma) in STEMdiff forebrain neuron differentiation media (StemCell Technologies) at a density of 125,000 cells/cm^2^. NPCs were maintained in culture for 7 days with daily media Change to achieve neuronal differentiation. On day 7, differentiated neurons were passaged to PLO/laminin-coated 6-well plates at a density of 80,000 cells/cm^2^ in STEMdiff forebrain neuron maturation media (StemCell Technologies). Neurons were cultured for 14 days with media changes performed every other day.

### Differentiation of astrocytes

Astrocytes were differentiated from NPCs using the STEMdiff astrocyte differentiation kit (StemCell Technologies). Briefly, NPCs were cultured in Matrigel-coated 6-well plates at a density of 1.5 × 10^5^ cells/cm^2^ in astrocyte differentiation media (StemCell Technologies). Cells were cultured for 7 days in STEMdiff astrocyte differentiation media with daily media change. Astrocyte precursor cells were passaged on days 7 and 14 and seeded in Matrigel-coated plates at a density of 1.5 × 10^5^ cells/cm^2^ in astrocyte differentiation media. On day 21, differentiated astrocytes were passaged into Matrigel-coated plates at a density of 1.5 × 10^5^ cells/cm^2^ in astrocyte maturation media (StemCell Technologies). Astrocytes were maintained in culture for 7 days with daily media change and then passaged twice at the same seeding density. Mature astrocytes are observed after 21 days in culture.

### Antibodies

Primary antibodies (all from Thermo Fisher): Zonula occludens 1 (ZO-1) (33-9100, 1:1000 dilution), P-glycoprotein (P-gp) (MA1-26528, 1:250 dilution), claudin-5 (35-2500, 1:500 dilution), glial fibrillary acidic protein (GFAP) (PA1-10004, 1:2000 dilution), microtubule-associated protein 2 (MAP2) (13-1500, 1:1000 dilution), beta 3 tubulin (50-4510-82, 1:1000 dilution), ionized calcium binding adaptor molecule 1 (IBA1) (10904-1-AP, 1:500 dilution), platelet-derived growth factor receptor beta (PDGFRβ) (MA5-15143, 1:500 dilution).

Secondary antibodies (all from Jackson Immuno Research): Alexa Fluor 488 goat anti-rabbit (111-545-003, 1:5000 dilution), Rhodamine (TRITC) goat anti-mouse (115-025-166, 1:2000 dilution), Alexa Fluor 647 donkey anti-chicken (703-605-155, 1:5000 dilution), Alexa Fluor 594 goat anti-rabbit (111-585-144, 1:2500 dilution), Alexa Fluor 488 donkey anti-mouse (715-454-150, 1:2500 dilution).

### Cellular characterization

Differentiation of hiPSC-derived cells was confirmed by immunocytochemistry. Differentiated cells were cultured in 8-well µ-slides (Ibidi), fixed with 4% paraformaldehyde (PFA), permeabilized with 0.2% tween20 and blocked with 1% bovine serum albumin (BSA). Characterization of iBMVECs was performed using antibodies against claudin-5, ZO-1, and P-gp. Pericytes were characterized using antibodies against PDGFRβ. Microglial cells were characterized with anti-IBA1 antibodies. Neurons were characterized using anti-MAP2, while astrocytes were characterized using anti-GFAP antibodies. Cells were then incubated with fluorescent secondary antibodies. Micrographs were acquired using a 20x objective in an inverted confocal microscope (Nikon ECLIPSE Ti2).

### Brain-chip seeding and culture

Differentiated cells were cultured on the Chip-S1 from Emulate Inc (Boston, MA USA). The Chip-S1 consists of two parallel fluidically independent microchannels made from polydimethylsiloxane (PDMS). The dimensions of the top channels are 1000 μm wide by 1000 μm height, and bottom channel’s dimensions are 1000 μm wide by 200 μm height. The overlapping co-culture area of the channels is 17.1 mm^2^. Channels are separated by a 50 μm porous PDMS membrane, with a 7 μm pore size and a 40 μm pore separation. Before culturing cells, the surface of the membranes was activated by introducing a mixture of the proprietary ER1 and ER2 reagents into the brain (top) and vascular (bottom) channels and exposing them to 2-cycles UV light irradiation for 10 min. The vascular channel of the chip was then coated with a mixture of 400 µg/mL human collagen IV and 100 µg/mL fibronectin in hECSFM. The brain channel was coated with 15 µg/mL PLO and 5 µg/mL laminin in BrainPhys media (StemCell Technologies). Chips were incubated overnight at 37 ℃ and 5% CO_2_. The following day, the vascular channel was washed with hECSR1 media and seeded with iBMVECs at a density of 8.5 × 10^5^ cells/cm^2^ in hECSR1 media supplemented with 10 µM Y-27632. Chips were inverted immediately after seeding and incubated at 37 ℃ and 5% CO_2_. Then, 2 hours later chips were returned to the upright position and sterile filter pipette tips were placed in the outlets of the channels and 200 µL of hECSR1 media were dispensed into the channels, leaving filter pipette tips on the inlet ports. Chips were returned to the incubator at 37 ℃ and 5% CO_2_ for overnight incubation. The following day, the vascular channel was washed with 200 µL of hECSR2 media, leaving filter tips in the inlet and outlet ports. Then, the brain channel was washed with 200 µL of proprietary microglia-forebrain neuron-astrocyte triculture media -referred to as brain media- (StemCell Technologies) that consisted of complete the STEMdiff forebrain neuron differentiation media supplemented with Microglia Supplement 2 (StemCell Technologies). After that, differentiated neurons (45,000 cells/cm^2^), astrocytes (20,000 cells/cm^2^), microglia (7,500 cells/cm^2^) and pericytes (12,500 cells/cm^2^) were pooled into a sterile microtube and seeded on the brain channel using brain media. Sterile filter tips were left in the inlet and outlet ports of the brain channel and chips were returned to the incubator at 37 ℃ and 5% CO_2_ for overnight incubation. The following day, both channels were washed with their respective media and chips were connected to the POD modules (Emulate Inc.). The modules consist of a chip-housing unit with reservoirs for cell culture media dispensing and effluent collection. Brain media was dispensed in the inlet reservoir of the brain channel and hECSR2 media was dispensed in the inlet reservoir of the vascular channel. Then, chips and PODs were connected to the Zoë Culture Module (Emulate Inc.), which was set to a flow rate of 20 µL/h for brain channel media and 60 µL/h for vascular channel media. Chips were incubated under flow conditions for 5 days, with daily media replenishment.

### Paracellular permeability

Paracellular permeability across the endothelial barrier was assessed by measuring the passage of 0.5 kDa Lucifer Yellow (LY) (Invitrogen), 3 kDa Dextran Cascade Blue (CB) (Invitrogen), and 10 kDa FITC-Dextran (Invitrogen) from the vascular channel media into the brain channel media by crossing the endothelial barrier. Tracers were dissolved in hECSR2 media at 20 µg/mL for 0.5 kDa LY, 100 µg/mL for 3 kDa Dextran CB and 10 kDa FITC-Dextran. Samples from the inlet and outlet reservoirs from both channels were collected daily for 5 days. Fluorescence intensity was read using a microplate reader (BioTek) using the following excitation/emission parameters: 485/528 nm for 10 kDa FITC-Dextran, 375/420 nm for 3 kDa Dextran CB, and 425/536 nm for 0.5 kDa LY. Apparent permeability was calculated using the following equation.

$$ \begin{aligned} Papp = & \frac{{Q_{R} *Q_{D} }}{{SA*\left( {Q_{R} + Q_{D} } \right)}} \\ & *ln\left[ {1 - \frac{{C_{{R,0}} *\left( {Q_{R} + Q_{D} } \right)}}{{\left( {Q_{R} *C_{{R,O}} + ~Q_{D} *C_{{D,O}} } \right)}}} \right] \\ \end{aligned} $$

where P_app_ is the apparent permeability in units of cm/s, SA is the surface area of the co-culture channel (0.17cm^2^), Q_R_ & Q_D_ are the fluid flow rates in the receiving and dosing channels, respectively, in units of cm^3^/s, and C_R,0_ and C_D,0_ are the recovered concentrations in the receiving and dosing channels, respectively. Three cell-free chips were used to obtain Papp values to use as reference.

### Immunocytochemistry analysis

Detection of cell-specific markers on the brain chip was assessed via immunocytochemistry. After 5 days in culture under flow conditions, chips were washed 3 times with DPBS and cells within both channels were fixed with 4% PFA, permeabilized with 0.2% tween20, and blocked with 1% BSA. The brain channel was incubated overnight at 4 ˚C with primary antibodies against MAP2, GFAP, IBA1 and PDGFRβ, while the vascular channel was incubated with antibodies against ZO-1. Chips were then incubated with fluorescent secondary antibodies for 2 h, counterstained with DAPI, and imaged using an inverted confocal microscope with a 10X magnification objective (Nikon ECLIPSE Ti2).

### Protein extraction

Protein was extracted from the brain and vascular channels of the brain-chips. After 5 days in culture, both channels were washed 3 times with 200 µL of sterile DPBS, which was aspirated after the last wash. Outlet ports of the chip were blocked with sterile pipette tips, then 60 µL of sterile DPBS was dispensed into the brain channel via the inlet port and 60 µL of complete radioimmunoprecipitation assay (RIPA) buffer (Thermo Scientific), supplemented with 1x protease and phosphatase inhibitors (Sigma) and 1 mM phenylmethylsulphonyl fluoride (Cell Signaling), were dispensed into the vascular channel. Chips were incubated at 4 ℃ for 30 min and sample was collected from the vascular channel. After that, DPBS was aspirated from the brain channel and replaced with 60 µL of RIPA buffer, while the vascular channel was filled with 60 µL of DPBS. Chips were incubated at 4 ℃ for 30 min and sample was collected from the brain channel. Protein concentration was then quantified by the bicinchoninic acid (BCA) method.

### ELISA

Protein samples (2.5 µg) from the vascular channel of the brain chips were used to quantify levels of the TJ protein claudin-5 (ABIN6962352), the membrane-bound receptor low-density lipoprotein receptor-related protein 1 (LRP-1) (ABIN6968348), and transporter P-gp (ABIN6958484) via ELISA kits from Antibodies Online. Kits were used according to manufacturer’s specifications and absorbance was read at 450 nm using a microplate reader. Protein values were interpolated in a standard curve and normalized by mg of protein.

### P-gp activity

Activity of the membrane-bound transporter P-gp was evaluated using the multidrug efflux transporter P-glycoprotein (MDR1/P-gp) ligand screening kit (ab284553) from Abcam. Briefly, media was aspirated from the brain and vascular channels and channels were washed twice with 100 µL warmed efflux assay buffer. Half of the chips were incubated with a fluorogenic P-gp substrate for 30 min at 37 ℃ and 5% CO_2_, while the other half was first incubated for 30 min with 100 µM of the P-gp inhibitor verapamil, before incubation with a mixture of 100 µM verapamil and 1x fluorogenic P-gp substrate at 37 ℃ and 5% CO_2_ for 30 min. Chips were then imaged using a Nikon ECLIPSE Ti2 confocal inverted microscope, under a stage-top incubator (Tokai Hit) maintaining physiological conditions of 37 ℃ and 5% CO_2_. The entire vascular channel of the brain-chips was imaged with a 10X objective and a FITC filter. Fluorescence intensity of the entire vascular channel was quantified using NIS Element software (Nikon).

### Quantification of amyloid beta and tau

The Milliplex Human Amyloid Beta and tau magnetic bead panel (Millipore Sigma, HNABTMAG-68k) was used to quantify levels of Aβ1–40, Aβ1–42, p-tau181, and total tau present within protein recovered from the vascular and brain channels (7.5 µg/protein lysate), as well as media effluent from both vascular and brain outlet reservoirs (pooled media from 5 days). The multiplex kits were used according to manufacturer’s specifications and plates were read using the BioPlex 200. The concentrations of Aβ1–40, Aβ1–42, p-tau181 and total tau were interpolated using a standard curve and normalized by mg of protein.

### Cytokine assessment

The BioPlex Pro Reagent Kit III (BioRad, 71304090 M) with a customized Human Cytokine screening panel was used to assess cytokines present in pooled media samples from vascular and brain effluent collected for 5 days. Magnetic beads targeting IL-6, TNF-α, IL-1β, MCP-1, IFN- γ, IL-12, IL-15 and IL-17a (BioRad, 17007269) were used. The multiplex kits were used according to manufacturer’s specifications and plates were immediately read using the BioPlex 200 using the same parameters as the MilliPlex kit.

### Data analysis

Data were obtained from two independent experiments that utilized cells from two independent differentiations. Each experiment consisted of *N* = 24 chips. Sample size for each individual endpoint is included in the respective figure legends. Data were analyzed using GraphPad Prism (v.10.0 for Windows, Boston MA USA). Normal distribution of the data was tested using the Shapiro-Wilk test. Comparisons of means of two experimental groups was performed using two-tailed *t*-test. For experiments with more than two groups, a one-way analysis of variance (ANOVA) was used followed by post-hoc Bonferroni multiple comparison test. For experiments with two factors, a mixed effect model was used to analyze the data, with hiPSC line (control and AD) and time (days) as fixed effects and individual chips as random effects, followed by Tukey’s test for multiple comparisons.

## Results

### Brain-chip model

**Fig. 1:**
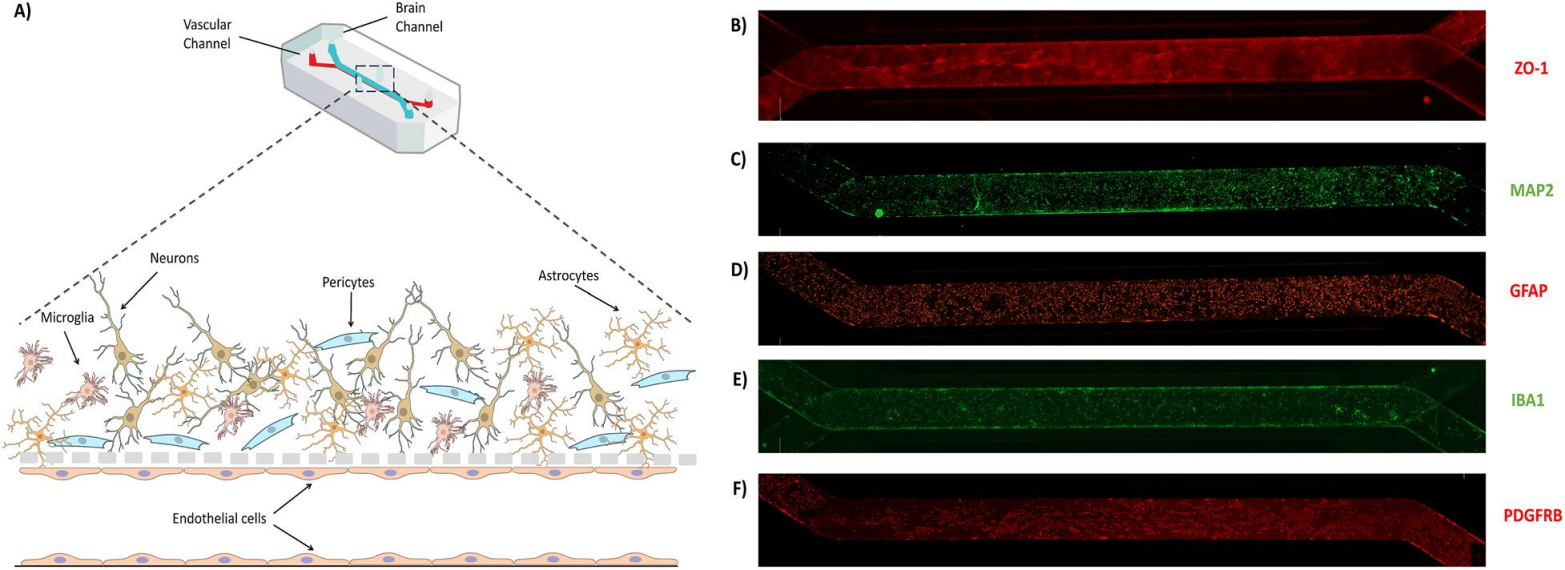
Brain-chip model. (A) Schematic representation of the brain-chip model. Neurons, astrocytes, microglia and pericytes are cultured on the top channel of the brain-chip, while iBMVECs are cultured on the bottom channel of the chip. Representative images of cells cultured on the brain-chip were obtained by immunocytochemistry. A monolayer of iBMVECs is observed by ZO-1 staining in the vascular channel (B). The presence of neurons (C), astrocytes (D), microglia (E) and pericytes (F) in the brain channel was confirmed by the cell-specific markers, MAP2, GFAP, IBA1 and PDGFRβ, respectively. Panel (A) was created using NIH BioArt

**Fig. 2:**
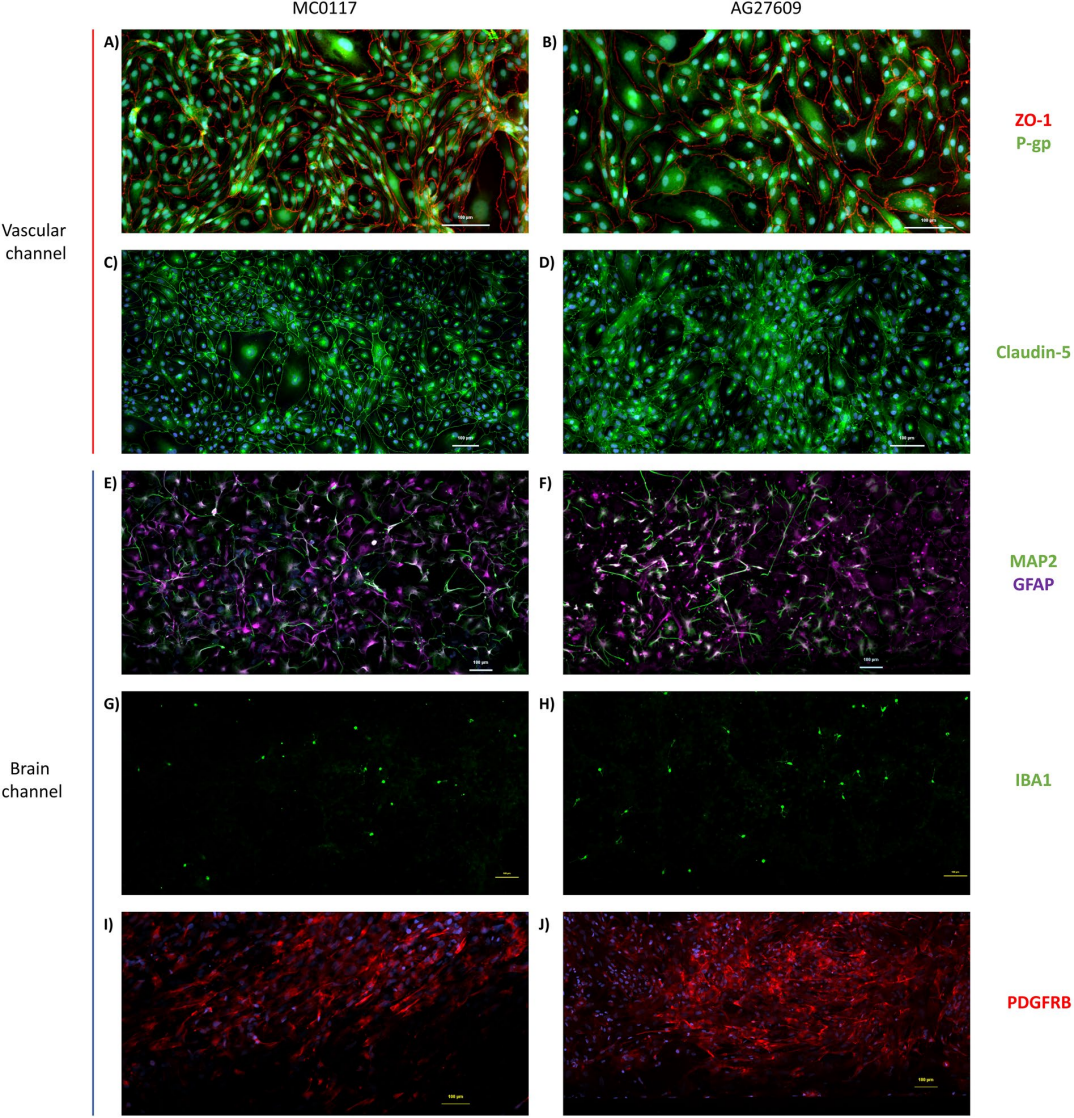
Morphological characterization of brain-chips. Confocal images of iBMVECs cultured on the vascular channel of the brain-chips showing the expression of ZO-1 and P-gp (A, B) and claudin-5 (C, D) in control and AD brain-chips. Confocal images of cells cultured on the brain channel of the chips showing neurons and astrocytes expressing MAP2 and GFAP respectively, (E, F); microglia expressing IBA1 (G, H); and pericytes expressing PDGFRβ (I, J) in control and AD brain-chips. Scale bar represents 100 μm

To construct the brain-chip model, hiPSCs were direct differentiated into neurons, astrocytes, pericytes, microglia and iBMVECs. Differentiation was confirmed by the expression of cell-specific markers for each cell type (Fig. S1). Differentiated iBMVECs were cultured on the vascular channel of brain-chips, while differentiated neurons, astrocytes, microglia, and pericytes were cultured on the brain channel of the chip (Fig. 1A). Homogeneous distribution of the cultured cells was observed along both channels. Formation of a continuous monolayer of iBMVECs on the vascular channel was confirmed by immunostaining with the TJ protein ZO-1 (Fig. 1B). The presence of neurons, astrocytes, microglia, and pericytes along the brain channel was confirmed by immunostaining with the cell specific markers MAP2 (Fig. 1C), GFAP (Fig. 1D), IBA1 (Fig. 1E) and PDGFRβ (Fig. 1F), respectively. There were no obvious morphological differences between cells differentiated from the hiPSC lines MC0117 (control) and AG27609 (AD) (Fig. 2), as evidenced by the staining patterns for the proteins ZO-1 (Fig. 2A and B), P-gp (Fig. 2A and B), claudin-5 (Fig. 2C and D), MAP2 (Fig. 2E and F), GFAP (Fig. 2E and F), IBA1 (Fig. 2G and H), and PDGFRβ (Fig. 2I and J).

### AD brain-chips present increased paracellular permeability related with a decrease in TJ proteins expression

**Fig. 3:**
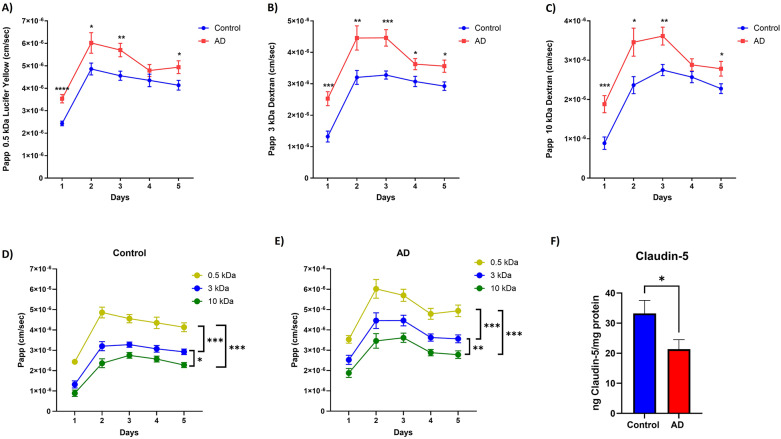
AD brain-chips present increased paracellular permeability to different size molecules. Apparent permeability (Papp) values for, 0.5 kDa lucifer yellow (A), 3 kDa cascade blue dextran (B) and 10 kDa FITC-dextran(C) for control and AD brain-chips during a 5-day period. Size-dependent permeability for the 3 different tracers for control (D) and AD (E) brain-chips is observed. Quantification of levels of the TJ protein claudin-5 (F) in protein lysates from the vascular channel of the brain-chips was performed by ELISA, values were normalized by mg of protein. Data represent the mean ± SEM. *N* = 18 chips/hiPSC line for Papp and *N* = 13 chips/hiPSC line for claudin-5 analysis. *p < 0.05

Paracellular permeability to different size molecules was assessed as a measure of BBB function during a 5-day period of culture under flow conditions. As expected, very high values of Papp were observed on cell-free chips, being 1.7 × 10^−5^, 1.85 × 10^−5^ and 2.09 × 10^−5^ for 10 kDa dextran, 3 kDa dextran and 0.5 kDa lucifer yellow, respectively. There was no interaction between hiPSC line (control and AD) and time for, 0.5 kDa lucifer yellow (Fig. 3A) [F (4, 127) = 1.066, *p* = 0.3763], 3 kDa dextran (Fig. 3B) [F (4, 127) = 1.280, *p* = 0.2814], or 10 kDa dextran (Fig. 3C) [F (4, 127) = 1.489, *p* = 0.2092]. Paracellular permeability increased overtime for 0.5 kDa lucifer yellow (Fig. 3A) [F (2.611, 82.89) = 37.84, *p* < 0.0001], 3 kDa dextran (Fig. 3B) [F (2.363, 75.03) = 26.96, *p* < 0.0001], and 10 kDa dextran (Fig. 3C) [F (2.240, 71.12) = 25.58, p = < 0.0001]. Notably, the increased permeability was only significant between days 1 and 2 for all tracers. There was a significant effect of hiPSC line (control vs. AD) on paracellular permeability for, 0.5 kDa lucifer yellow (Fig. 3A) [F (1, 32) = 10.85, *p* = 0.0024], 3 kDa dextran (Fig. 3B) [F (1, 32) = 35.49, *p* < 0.0001] and 10 kDa dextran (Fig. 3C) [F (1, 32) = 21.12, p = < 0.0001], with AD brain-chips presenting a significant increase in permeability for the 3 tracers at all time points, with the exception of day 4, for 0.5 kDa lucifer yellow (*p* = 0.2658) and 10 kDa dextran (*p* = 0.1487). Analysis within hiPSC line demonstrated size-dependent permeability for both, control brain-chips (Fig. 3D) [F (2, 45) = 67.75, *p* < 0.0001)] and AD brain-chips (Fig. 3E) [F (2, 51) = 39.40, *p* < 0.0001)], with higher permeability values observed for lower molecular weight tracers. Alterations in paracellular permeability are often related to decreased expression of TJ proteins. Therefore, we quantified levels of the TJ protein claudin-5 in protein lysates from the vascular channel of the brain-chips (Fig. 3F). We observed a decrease in claudin-5 expression in AD brain-chips compared to control brain-chips (*p* = 0.0230). Expression of ZO-1 was also observed in AD brain-chips compared to control brain-chips (*p* = 0.0103) (Supplementary Fig. 2). These data indicate that AD brain-chips present increased paracellular permeability to different size molecules that was related with a decrease in the expression of TJ proteins.

### AD brain-chips present decreased expression and activity of proteins related to Aβ clearance

**Fig. 4:**
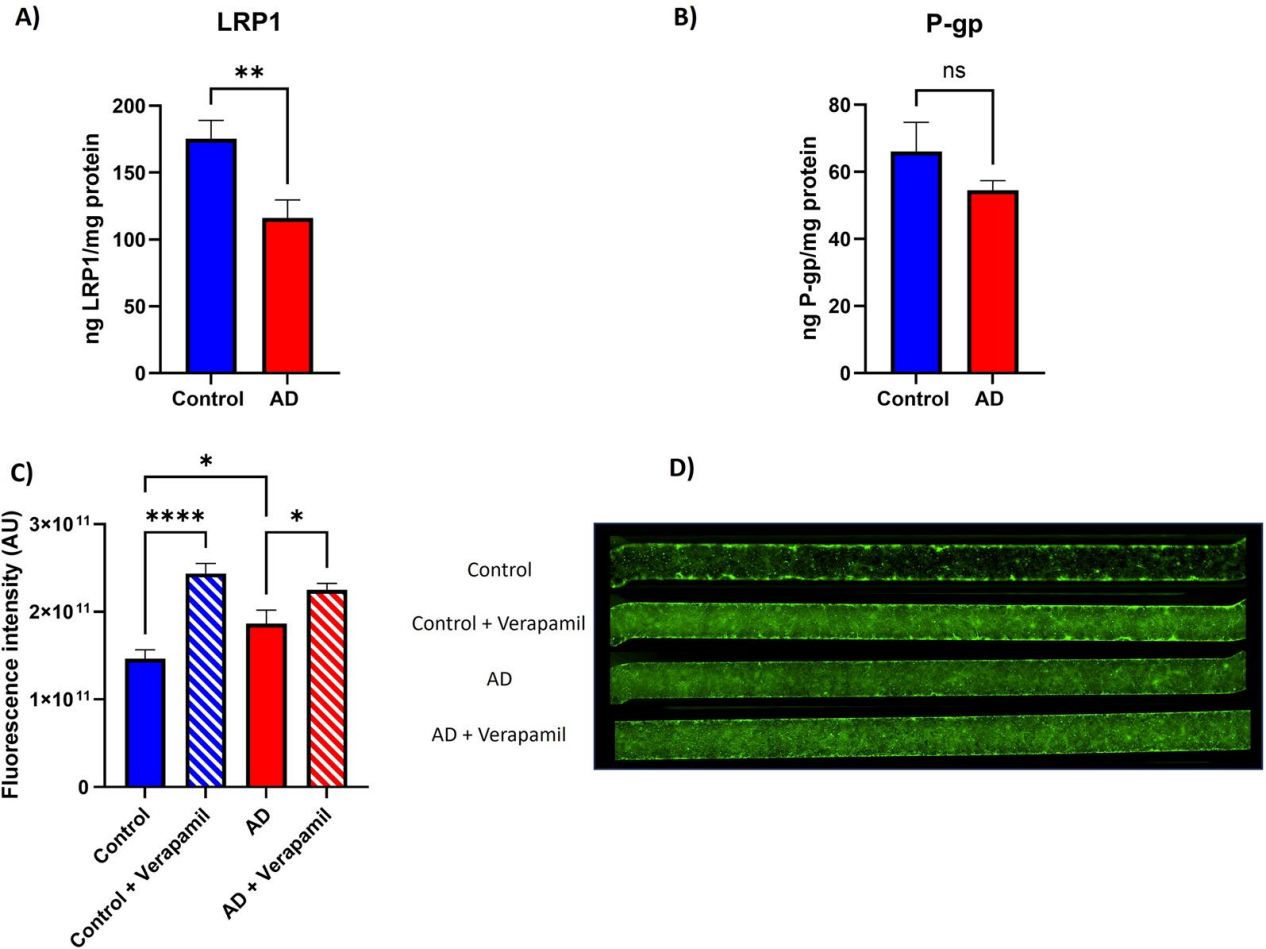
Expression and activity of proteins associated with Aβ transport is decreased in AD brain-chips. Quantification of levels of LRP1 (A) and P-gp (B) in protein lysates from the vascular channel of the brain-chips was performed by ELISA, values were normalized by mg of protein. Activity of P-gp (C) was evaluated by analyzing the fluorescence intensity of a fluorescent P-gp substrate on the vascular channel of the chip, the P-gp inhibitor verapamil was used as positive control. Representative micrographs from the vascular channel of the brain-chips showing the fluorescent signal of the P-gp substrate (D). Data represent the mean ± SEM. *N* = 13 chips/hiPSC line for LRP1 and P-gp ELISA. *N* = 5 chips/condition for P-gp activity analysis. *p < 0.05, **p < 0.01, ****p < 0.0001

Because impaired Aβ clearance is implicated in AD, we quantified levels of Aβ transporters LRP1 (Fig. 4A) and P-gp (Fig. 4B) in protein lysates from the vascular channel. We found decreased expression of LRP1 (Fig. 4A) in AD brain-chips, compared to control brain chips (*p* = 0.0031), while the expression of P-gp remained unchanged (*p* = 0.1342). Then, we assessed the activity of P-gp on iBMVECs cultured on the vascular channel. To that end, we inhibited P-gp function by incubating the vascular channel of the brain-chips with 100 µM verapamil and then adding a fluorescent P-gp substrate that will diffuse into the iBMVECs. When functioning properly, P-gp will actively transport the substrate out of the cells, leading to a decrease in fluorescence signal (Fig. 4C and D). Activity of P-gp was decreased in AD brain-chips, compared to control (*p* = 0.0334). As expected, verapamil decreased P-gp activity, compared to untreated chips, in both control (*p* < 0.0001) and AD-brain chips (*p* = 0.0313), although the difference was smaller in AD brain-chips. These data suggest that mechanisms involved in Aβ clearance are altered in AD brain-chips from this particular donor.

### Different expression of Aβ and tau are observed in brain and vascular samples from AD brain-chips

**Fig. 5:**
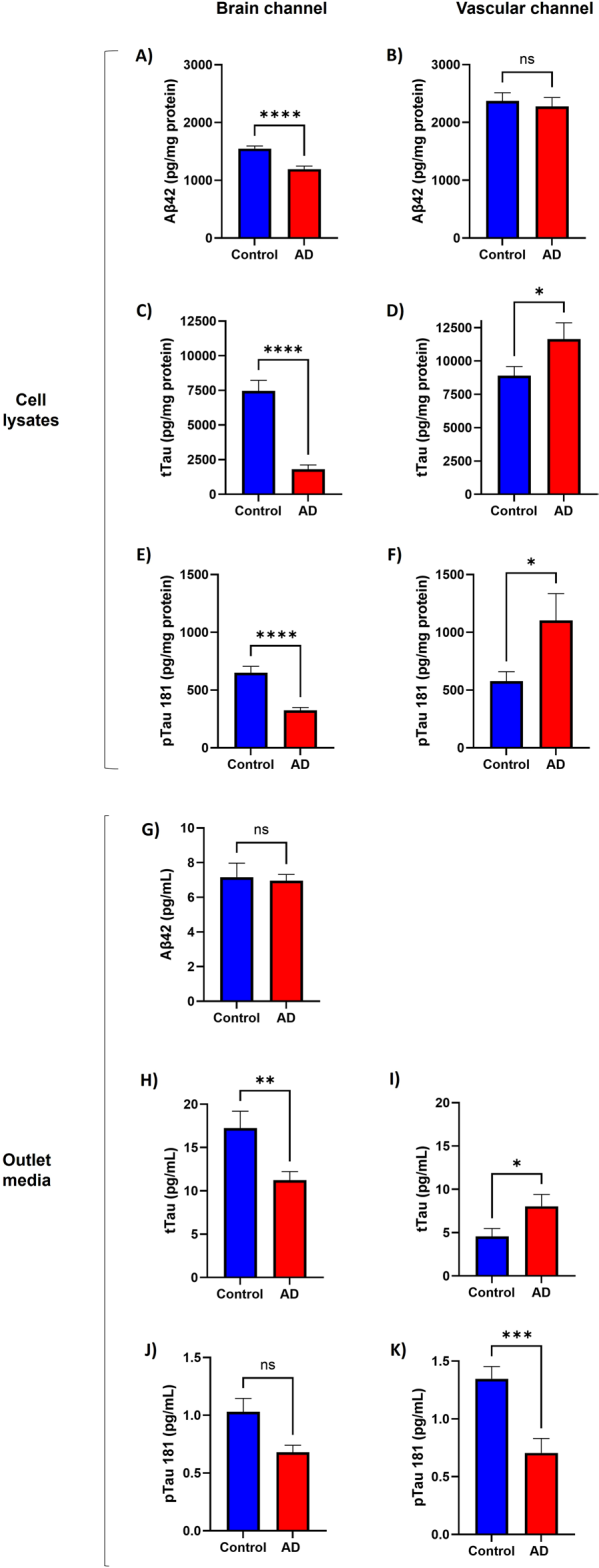
Lower levels of brain Aβ and tau, and higher levels of vascular tau are observed in AD brain-chips. Levels of Aβ42 (A, B, G); total Tau (C, D, H, I); and pTau 181 (E, F, J, K) were quantified in cell lysates (A-F), and pooled effluent media (G-K), from the brain and vascular channels of the brain-chips. Data represent the mean ± SEM. *N* = 13 chips/hiPSC line. *p < 0.05, **p < 0.01, ***p < 0.001, ****p < 0.0001

Pathological hallmarks of AD include increased production of Aβ and tau. We quantified the amount of Aβ40, Aβ42, total tau and pTau 181 in protein lysates and cell culture media from the brain and vascular channels of the brain-chips. Aβ40 levels were below the limit of detection for all samples analyzed. Surprisingly, we observed decreased levels of Aβ42 (Fig. 5A, *p* < 0.0001), total tau (Fig. 5C, *p* < 0.0001) and pTau 181(Fig. 5E, *p* < 0.0001) in protein lysates obtained from the brain channel of AD brain-chips. Levels of total tau were also decreased in the media effluent of AD brain-chips compared to control (Fig. 5H, *p* = 0.0080), while the levels of Aβ42 (Fig. 5G) and pTau 181 (Fig. 5J) were not different between the two groups. On the other hand, a completely different pattern on the levels of these proteins was observed in samples obtained from the vascular channel of the brain-chips. We observed a significant increase in the levels of total tau (Fig. 5D, *p* = 0.02750) and pTau 181 (Fig. 5F, *p* = 0.0331) in protein lysates from the vascular channel of AD brain-chips. Levels of total tau were increased (Fig. 5I, *p* = 0.0476), while pTau 181 (Fig. 5K, *p* = 0.0009) were decreased in effluent media collected from the vascular channel of AD brain-chips. For Aβ42, there was no difference between the two groups on protein lysates from the vascular channel (Fig. 5B, *p* = 0.6745) and its values were below the limit of detection for effluent media from the vascular channel. These data indicate that brain-chips constructed with hiPSCs from this AD patient have lower levels of Aβ and tau in the brain channel, although vascular tau accumulation was detected.

### AD brain-chips have increased levels of proinflammatory markers

**Fig. 6:**
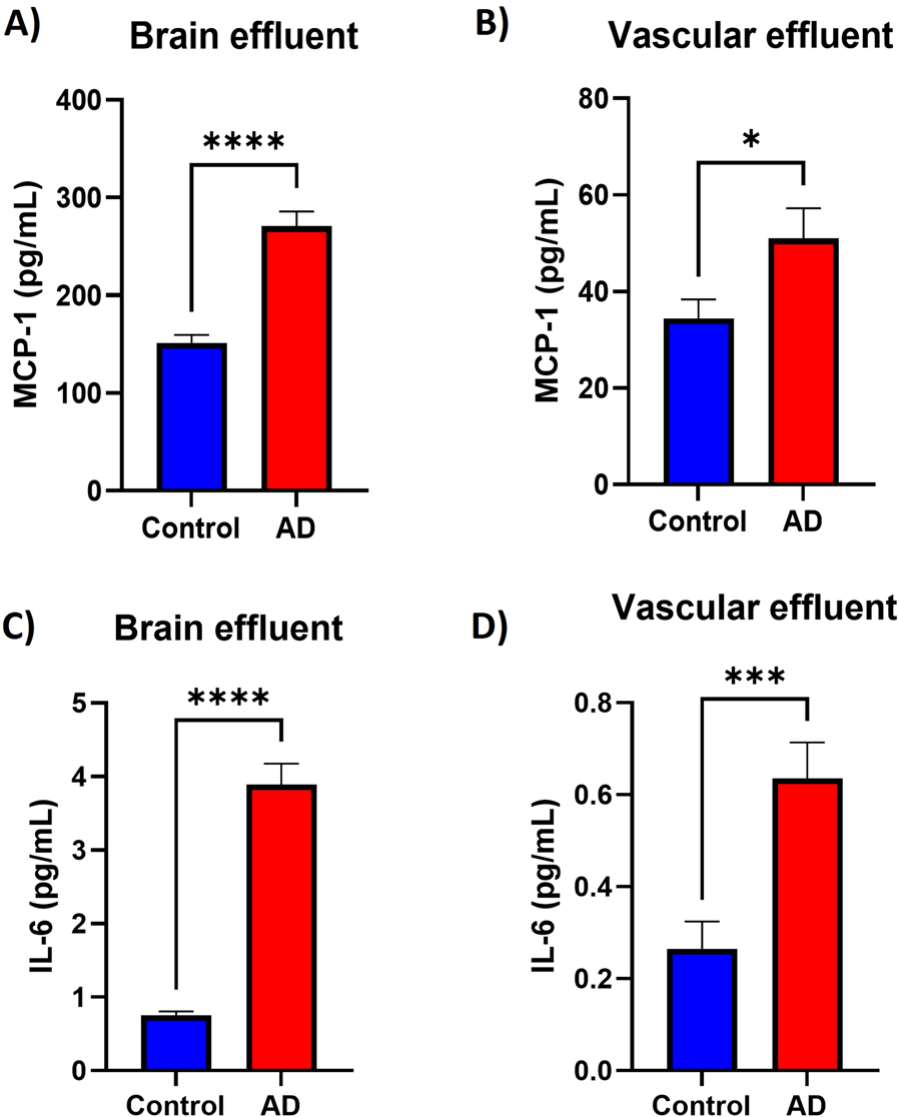
AD brain-chips secrete proinflammatory markers. Levels of the inflammatory markers MCP-1 (A, B) and IL-6 (C, D) were quantified in pooled media effluent from the brain (A, C) and vascular (B, D) channels. Data represent the mean ± SEM. *N* = 13 chips/hiPSC line. *p < 0.05, ***p < 0.001, ****p < 0.0001

Neuroinflammation is also recognized as a contributing factor to AD pathology. We quantified levels of inflammatory markers in effluent media from the brain and vascular channels of the brain-chips. Levels of TNF-α, IL-1β, IFN-γ, IL-12, IL-15 and IL-17a were below the limit of detection for all samples analyzed. We observed increased levels of MCP-1 on brain (Fig. 6A, *p* < 0.0001) and vascular (Fig. 6, *p* = 0.0365) effluents from AD brain-chips. Similarly, levels of IL-6 were also increased in brain (Fig. 6C, *p* < 0.0001) and vascular (Fig. 6D, *p* = 0.0009) effluents from AD brain-chips. These data suggest that increased production of some neuroinflammatory markers was observed in AD brain-chips for this specific hiPSC line.

## Discussion

Neurovascular dysfunction is recognized as one of the pathological hallmarks of AD [2, 49] and usually occurs before the onset of cognitive decline [4]. Studies have demonstrated decreased expression of TJ proteins is associated with increased BBB permeability [5, 50, 51] and impairment of the clearance mechanism of Aβ from the brain, which includes decreased expression of P-gp in cases of AD [7, 52] and other instances of cognitive decline [53, 54].

Here, we characterized neurovascular functions associated with AD pathology in brain-chips constructed from hiPSCs obtained from a healthy individual with the ε3 allele of the APOE gene, and a patient with late-onset AD that carried the ε4 allele of APOE. On AD brain-chips, we described decreased expression of TJ proteins, associated with increased BBB permeability and neuroinflammation, that was related to an increase in vascular tau accumulation and independent of Aβ production.

Previous studies have differentiated iBMVECs from hiPSCs obtained from patients with familial forms of AD and characterized their phenotypes. For instance, iBMVECs differentiated from hiPSCs from an AD patient with a mutation in PSEN1 had decreased expression of TJ proteins, associated with increased permeability. In addition, impaired activity of efflux transporters, including P-gp was observed [35, 55]. In AD brain chips, we observed increased paracellular permeability, decreased expression of TJ proteins, and reduced activity of an efflux transporter partly responsible for clearance of Aβ from the brain. Studies using iBMVECs derived from hiPSCs from healthy individuals and patients with sporadic AD that carry the ε4 allele of APOE have also been conducted. It is worth mentioning that iBMVECs obtained from hiPSCs using multiple differentiation protocols, including the one used in this study [25], may present different phenotypes due to particular differentiation processes and interpretation of the results obtained using these cells may not fully replicate the behavior of brain microvascular endothelial cells [56]. However, iBMVECs obtained using these protocols can still be used to model different aspects of the cerebral vasculature. For instance, one study used iBMVECs that were differentiated from isogenic hiPSCs lines genetically modified to express either the ε2, ε3 or ε4 alleles of APOE. Presence of the ε2 and ε3 alleles was associated with increased clearance of Aβ42 and Aβ40 peptides, respectively; however, there was no difference in paracellular permeability and the expression of TJ proteins or efflux transporters between all lines [34]. On the contrary, a different study found an increase in permeability and altered expression of TJ in iBMVECs differentiated from hiPSCs with the ε4 allele of APOE, compared to ε3 [57]. A more comprehensive study used hiPSC from an AD patient (ε4 allele of APOE), and a healthy subject (ε3 allele of APOE) to differentiate iBMVECs, pericytes, astrocytes, microglia, and NPCs to produce multiple co-culture models of iBMVECs with individual cells using a transwell model. The authors found that co-cultures of iBMVECs with NPCs, but not other cell types, increased barrier properties only in control, but not AD co-cultures. However, alterations in gene expression of TJ proteins, ABC transporters, including P-gp and lipoprotein receptors, including LRP1 were observed in the AD model [58], suggesting endothelial-specific alterations. Here we also used hiPSCs-derived brain cells from an AD patient with the ε4 allele of APOE, and a healthy subject with the ε3 allele to construct a more physiologically relevant model of the NVU. Instead of using a transwell system to produce different co-cultures, we cultured together all five cell types that form the NVU in an organ-chip to produce a model that more closely resembles the brain vasculature. Like previous studies, we observed size-dependent increase in paracellular permeability coupled with decreased expression of TJ proteins in AD brain-chips. We also observed a decrease in endothelial levels of LRP1 in the AD brain-chips but found no difference in the levels of P-gp between the two groups. Despite no change in P-gp expression, P-gp activity was decreased in AD brain-chips, indicating alterations in one of the mechanisms of Aβ elimination.

Rodent models of AD have shown decreased P-gp expression and activity in brain capillaries, compared to wild type animals [59–62]. Additional studies have reported decreased expression of P-gp and LRP1 in normal aging in rodents, with decreases in LRP1 expression preceding decreases in P-gp [63, 64]. Expression [52] and activity [65] of P-gp are both decreased as a function of age in humans, and both have been associated with increased deposition of Aβ peptides [52]. Postmortem analysis of brains of patients with AD have shown decreased P-gp expression in brain microvessels compared to age-matched controls [66]. Yet, other studies have not found a difference in P-gp expression but that does not rule out reduced P-gp activity [67]. Multiple studies analyzing P-gp function using the PET tracer [^11^C]verapamil have shown decreased activity in multiple brain regions in patients with AD compared to age-matched controls [7, 68], which in turn can lead to accumulation and aggregation of Aβ peptides. On the contrary, we observed decreased levels of Aβ42 in lysates from the brain channel of AD-chips compared to control and found no difference in Aβ42 levels in lysates from the vascular channel and media effluent from the brain channel between groups. Previous studies using hiPSCs-derived neurons from AD patients have shown increased production and secretion of Aβ peptides [20, 26, 28, 31, 69]. However, multiple studies have reported high variability in the production and secretion of Aβ peptides by neurons differentiated from hiPSCs obtained from AD patients [70]. This variability, likely driven by genetic differences, highlights an important limitation in the use of single hiPSCs lines within a particular study.

We also observed lower expression levels of total tau and pTau 181 in lysates from the brain channel of AD-chips, which was also inconsistent with AD pathology [1, 71]. Increased levels of phosphorylated forms of tau including tau 181 have been observed in neurons differentiated hiPSCs derived from sporadic and familial AD cases [72, 73]. Levels of total tau were also decreased in effluent media from the brain channel of AD brain-chips. However, we observed increased levels of total Tau and pTau 181 in protein lysates of the vascular channel of the AD brain-chips. It is possible that tau produced by cells on the brain channel may accumulate in iBMVECs on the vascular channel, which resulted in the observed vascular dysfunction in AD brain-chips. Depositions of tau oligomers have been observed in the brain vasculature in humans, as well as animal models of AD [74, 75]. Results from in vitro studies suggest that fibrillary tau increases BBB permeability in human endothelial cells, associated with decreased expression of TJ proteins and an increase in proinflammatory markers [76]. A recent study using a rodent model of tauopathy reported that pathogenic forms of tau can enter brain endothelial cells and induce vascular dysfunction, associated with an increase of neuroinflammatory markers, including IL-6 and MCP1 [77], which have been reported to increase BBB permeability [78–80]. Similarly, we report increased IL-6 and MCP1 expression in brain and vascular media effluents of AD brain-chips, which may be a consequence of tau accumulation in brain endothelial cells that contributed to increased BBB permeability.

Our results support a link between NVU dysfunction and tau accumulation on iBMVECs, but the presence of the ε4 allele of APOE may also play a role in the observed phenotype. Indeed, seminal work by Montagne and colleagues (2020) [81] found that the mere presence of the ε4 allele, independent of Aβ and tau accumulation, contributes to BBB dysfunction in humans. This finding has been corroborated by multiple studies [54, 82, 83]. Additional studies have found that, among AD patients, NVU dysfunction and increased BBB permeability are aggravated by the ε4 allele relative to ε3 allele carriers [84, 85]. The ε4 allele has also been associated with increased tau production [86, 87]. Given we used AD donor cells from an APOE ε4 carrier and observed NVU dysfunction, our results are in line with previous findings.

Despite recent advances in the use of hiPSC-derived cells for the modeling of neurodegenerative disorders [88], including AD [89, 90], there are several limitations with these models [91]. Such limitations include the relatively short periods of time that can be maintained in culture, failing to model ageing [92] and the loss of epigenetic signatures that can affect AD phenotype, as studies have reported that direct differentiation of fibroblasts into neurons present epigenetic profiles that resemble human brain ageing, compared to neurons differentiated from hiPSCs [93]. However, hiPSC-derived models of neurodegenerative disorders can still be useful in understanding different aspects of these pathologies [88, 90].

This study also serves to highlight limitations associated with the use of individual patient-derived hiPSC lines. Due to the genetic heterogeneity between different donors, cells derived from multiple hiPSCs lines can present different phenotypes [94], i.e., an AD donor cell line may express less Aβ42 than a cognitively normal donor cell line. A recent study aimed to address optimal study designs and sample sizes to obtain adequate statistical power in studies using hiPSC lines estimates that many studies are underpowered and do not use an adequate sample size [95]. In addition, the use of a single, or a few hiPSC lines fails to include genetic variability and can be less predictive of the response of a population of interest. Different strategies have been used to address this issue, for instance, a recent study has proposed using the expression of high-frequency human leukocyte antigen (HLA) haplotypes to represent a high percentage of the Asian population. Using this approach, the authors identified 13 hiPSC lines and tested neurotoxic and cardiotoxic compounds in neurons and cardiomyocytes differentiated from these population-based hiPSCs and captured specific responses to neurotoxic and cardiotoxic compounds based on the HLA haplotype [96]. Although testing cells derived from multiple hiPSC lines can increase the predictive validity of a given study, it also increases the number of experiments that need to be performed. Another interesting approach to conduct population-scale hiPSC studies is the use of “village in a dish” cultures, consisting of multiple hiPSC lines cultured and differentiated together [97]. This model allows for the introduction of individual variability in experiments using hiPSCs, without significantly increasing the workload, as cellular differentiation and endpoints for all lines are performed simultaneously in the village cultures. Similarly, recent studies have used brain organoids obtained from multiple hiPSC lines differentiated together, using either the term “brain chimeroids” [98] or “mosaic brain organoids” [99]. Using these methods, the effects of neurotoxic compounds can be assessed in multiple lines simultaneously, in addition, individual responses can also be analyzed using single-cell RNA-sequencing, providing additional data on specific responses within a population of interest [99]. These or similar approaches can be adapted in the construction of brain-chips to evaluate NVU functions related to AD to capture responses of multiple individuals that may be more predictive of population responses.

## Conclusions

In summary, using hiPSC-derived cells of the NVU cultured in an MPS, we were able to construct a brain-chip model to assess neurovascular functions related with AD pathology. Using these specific hiPSC lines, we demonstrated increased paracellular permeability and neuroinflammation, that were independent of Aβ production and related with vascular tau accumulation. Models including cells differentiated from multiple hiPSCs from AD patients can be constructed and characterized to include individual variabilities within the AD population and may be useful in determining genetic predispositions to neurovascular dysfunction. Complete characterization and validation of these models will be necessary before they can be used with confidence in initial evaluations of safety and efficacy of novel therapies being developed for AD.

## Supplementary Information

Supplementary Material 1.

## Data Availability

Data is provided within the manuscript or supplementary information files.

## Abbreviations

AD: Alzheimer’s disease
ANOVA: Analysis of variance
APP: Amyloid precursor protein
Aβ: Amyloid beta
BBB: Blood-brain barrier
BCA: Bicinchoninic acid
bFGF: Basic fibroblast growth factor
BSA: Bovine serum albumin
CB: Cascade blue
GFAP: Glial fibrillary acidic protein
hECSFM: Human endothelial cell serum free media
hiPSCs: Human induced pluripotent stem cells
HLA: Human leukocyte antigen
IBA1: Ionized calcium binding adaptor molecule 1
iBMVECs: Induced brain-like microvascular endothelial cells
IFN-γ: Interferon gamma
IL-12: Interleukin 12
IL-15: Interleukin 15
IL-17a: Interleukin 17a
IL-1β: Interleukin 1 beta
IL-6: Interleukin 6
LRP-1: Low-density lipoprotein receptor-related protein 1
LY: Lucifer yellow
MAP2: Microtubule-associated protein 2
MCP-1: Monocyte chemoattractant protein-1
MPS: Microphysiological systems
NAMS: New alternative methods
NIM: Neural induction media
NPCs: Neural progenitor cells
NVU: Neurovascular unit
PDGF-BB: Platelet-derived growth factor BB
PDGFRβ: Platelet-derived growth factor beta
PDMS: Polydimethylsiloxane
PFA: Paraformaldehyde
P-gp: P-glycoprotein
PLO: Poly-L-ornithine
PMPs: Primitive macrophage precursor cells
PSEN2: Presenilin-2
PSEN1: Presenilin-1
RIPA: Radioimmunoprecipitation assay
TNF-α: Tumor necrosis factor alpha
ZO-1: Zonula occludens 1
