# Supplementary material for: Modeling neurovascular dysfunction in Alzheimer’s disease using an isogenic brain-chip model
Source: Fluids Barriers CNS. 2026 Jan 6;23:1. doi: 10.1186/s12987-025-00708-y (PMC12777506; doi:10.1186/s12987-025-00708-y)
Supplement: Supplementary file 1 — Supplementary Material 1. [file 12987_2025_708_MOESM1_ESM.docx]

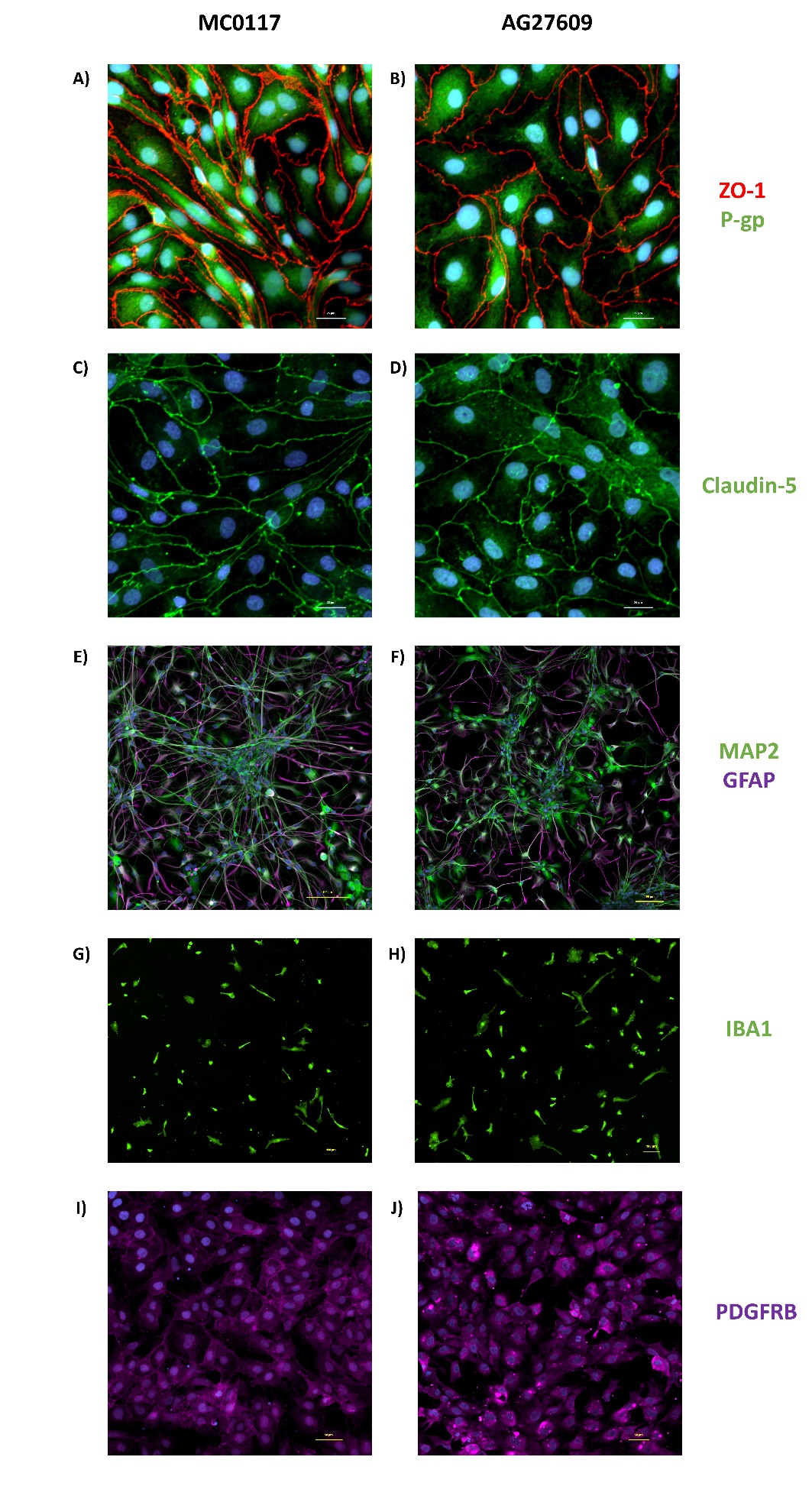


**Supplementary Figure 1**

**Confocal images of hiPSC derived cells cultured on 8-well µ-slides**. Immunocytochemical analysis of iBMVECs showing the expression of ZO-1 and P-gp (A, B) and claudin-5 (C, D) in control (MC0117) and AD (ag27609) cells. Neurons and astrocytes expressing MAP2 and GFAP respectively, (E, F); microglia expressing IBA1 (G, H); and pericytes expressing PDGFRβ (I, J) in control and AD cells.


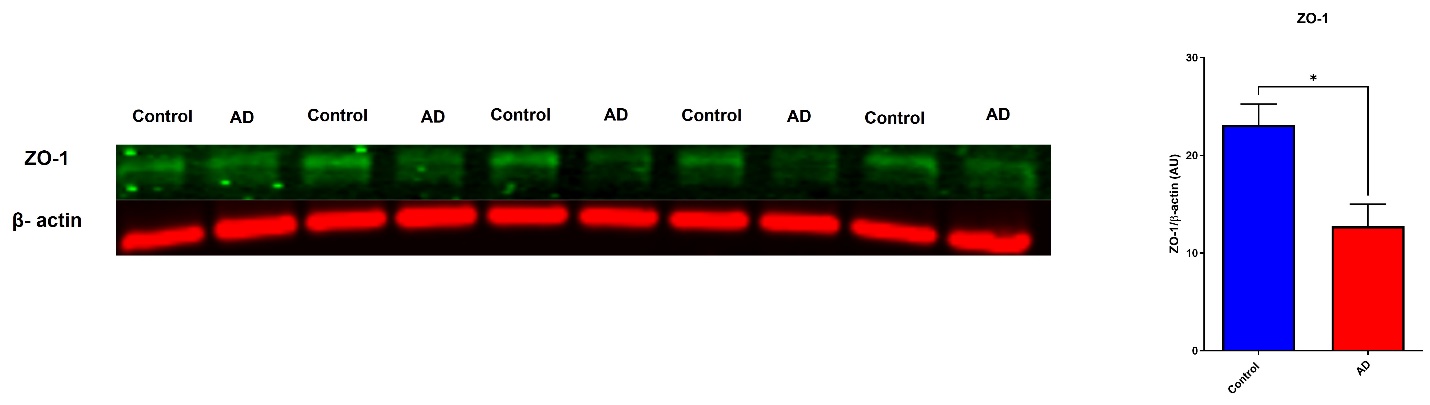


**Supplementary Figure 2**

**Western blot analysis of ZO-1 expression in protein lysates from the vascular channel of control and AD brain-chips.** Five µg of protein of vascular channel lysate were loaded into Sodium Dodecyl Sulfate Polyacrylamide Gel Electrophoresis (SDS-PAGE) gels and then transferred to polyvinylidene fluoride (PVDF) membranes. Membranes were incubated overnight with antibodies against ZO-1 (Thermo Fisher: 33-9100, 1:1000 dilution) and β-actin (Millipore Sigma A2228, 1:2500 dilution) and then incubated for 1 h with secondary antibodies (Licor: 926-32212 IRDye® 800CW and 926-68072 IRDye® 680RD, 1:20,000 dilution). Images were acquired using a LiCor Odyssey imaging system and the intensity of the bands was analyzed using Image Studio V 5.0. Values of ZO-1 were normalized to the loading control (β-actin). *p<0.05. N= 5 chips/line.


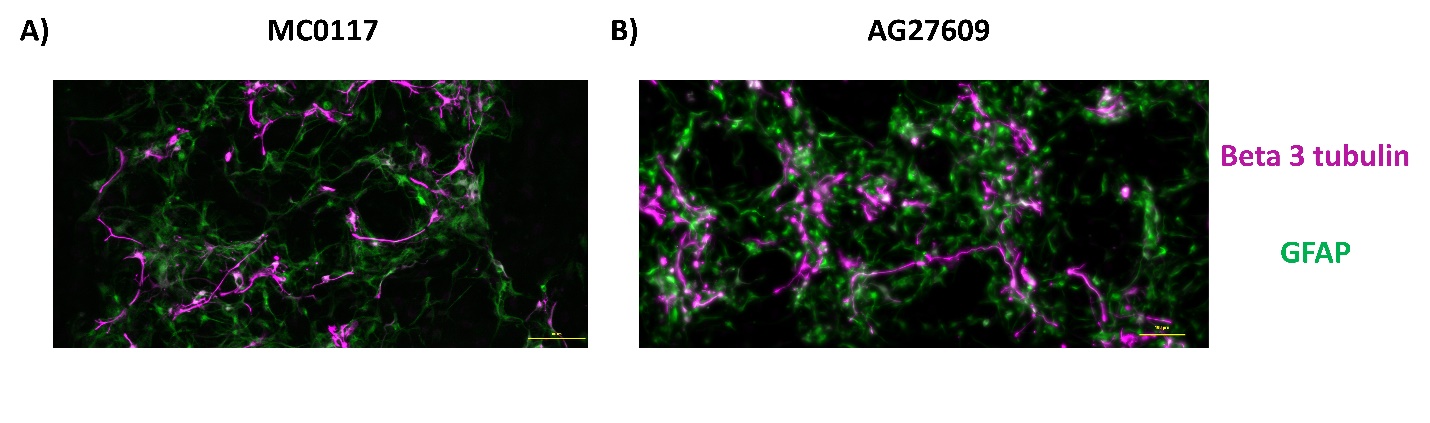


**Supplementary Figure 3**

**Expression of neuronal and astrocytic markers**. Confocal images of cells cultured on the brain channel of the chips showing neuronal expression of beta 3 tubulin (magenta) and astrocytic expression of GFAP (green) in A) control and B) AD brain-chips. Scale bar represents 100 µm.
